# Detection of Temperature Difference in Neuronal Cells

**DOI:** 10.1038/srep22071

**Published:** 2016-03-01

**Authors:** Ryuichi Tanimoto, Takumi Hiraiwa, Yuichiro Nakai, Yutaka Shindo, Kotaro Oka, Noriko Hiroi, Akira Funahashi

**Affiliations:** 1 Keio University, Department of Biosciences and Informatics, 3-14-1, Hiyoshi, Kohoku-Ward, Yokohama, 223-8522, Japan

## Abstract

For a better understanding of the mechanisms behind cellular functions, quantification of the heterogeneity in an organism or cells is essential. Recently, the importance of quantifying temperature has been highlighted, as it correlates with biochemical reaction rates. Several methods for detecting intracellular temperature have recently been established. Here we develop a novel method for sensing temperature in living cells based on the imaging technique of fluorescence of quantum dots. We apply the method to quantify the temperature difference in a human derived neuronal cell line, SH-SY5Y. Our results show that temperatures in the cell body and neurites are different and thus suggest that inhomogeneous heat production and dissipation happen in a cell. We estimate that heterogeneous heat dissipation results from the characteristic shape of neuronal cells, which consist of several compartments formed with different surface-volume ratios. Inhomogeneous heat production is attributable to the localization of specific organelles as the heat source.

Temperature is one of the most important parameters of the intracellular environment because it changes the dynamics and the reactivity of biomolecules^1^. Each biological reaction that controls cell functions occurs either exothermically or endothermically in particular microdomains of a cell. Biological reactions cause the release of Gibbs free energy, which triggers cellular functions. The unused energy is transformed into heat; the heat production then elevates the local temperature. Proton transfer through the mitochondrial inner membrane is a mechanism of thermogenesis and acts as a heat source in a cell^2^. The endoplasmic reticulum (ER) also produces heat via calcium pump activity^3^. These mechanisms are the assumed scenarios for any increase in temperature locally, as a result reaction rates are accelerated following the change in chemical equilibria. Therefore, if there exists a heterogeneous temperature distribution within a cell, it may strongly correlate with cell activities via biochemical reaction processes. The temperature distribution within a cell measured in the previous studies may be too small to affect the cellular functions, which has not been indicated a strong correlation between their shapes and the functions, such as with fibroblast^4^,^5^. However, it can play significant roles in intricately shaped cells, for example, neuronal cells, because cell body can have a larger volume divided by a surface area compared to neurites. The energy exchange between intra- and extracellular domains is also thought to show asymmetric patterns dependent on both spatial and functional asymmetric properties of the cells.

Fluorescence-based imaging has been the main strategy for intracellular thermometry^6^,^7^,^8^, because it can be non-invasive and can have a high spatiotemporal resolution compared with a classical calorimetry^9^ or infrared thermography^10^. EuTTA^11^,^12^,^13^, fluorescent proteins^14^,^15^, gold nanoclusters^16^, nanodiamonds^17^, small molecules targeting a specific organelle^18^,^19^, polymer nanoparticles^5^,^20^,^21^, quantum dots^4^,^22^, and silica nanoparticles^23^ are utilized as thermosensors in combination with several optical arrangements. Temperature measurement is achieved by the appropriate combination of thermometric properties and thermosensors. Fluorescence imaging-based thermometry utilizes the temperature dependent change of emission intensity, emission lifetime, or emission wavelength at maximum intensity. These techniques have revealed the temperature distribution^5^ or event-driven temperature elevation in a cell^4^,^13^,^15^.

These previous works suggest that many biochemical reactions in a cell are influenced by the local heat production and heterogeneity of temperature. In particular, the reactions and hence the functions of structurally complicated cells such as neuronal cells are likely to be related to differences in temperature. However, there have been no reports on the temperature difference in neuronal cells, and there is no definite information about how the heterogeneity of temperature is formed. In order to detect the subtle temperature differences in a cell which should maintain the *in vivo* environment, non-invasive measurement, non-localization and stable temperature sensitivity of thermosensors are important factors. Taking these requirements into account, existing thermometry using quantum dot-based nanoparticles is thought to be suitable, as it satisfies these requirements. Quantum dots have broader excitation wavelengths and brighter fluorescence than other thermodetectors^24^. This makes them easy to detect, although their erratic behaviour sometimes presents problems^4^. However, detecting their temperature dependent shift of emission wavelength at maximum intensity requires a spectrograph with a high resolution of grating, as suggested above^25^.

In this paper, we developed a novel method for both sensing intracellular time-lapse temperature change, and spatial temperature difference by utilizing commercially available quantum dot-based nanoparticles and a confocal microscope. We demonstrate how to detect the temperature increase after the mitochondrial stimulation with the sequential observation of single quantum dot, and for the first time the temperature difference within a neuronal cell by using multiple quantum dot imaging, and suggest the cause of heterogeneity of temperature by using the characteristic shape of neuronal cells based on the actual 3D observation.

## Results

### Quantum dot-based ratiometric thermometry

**Figure 1:**
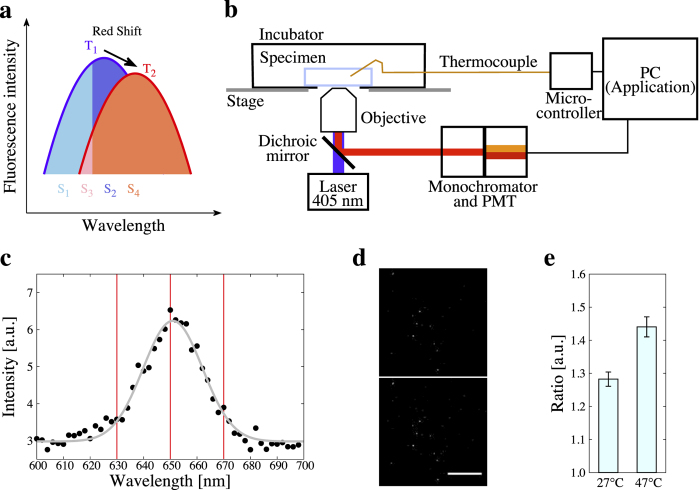
Experimental outline. (**a**) Schematic diagram of a single fluorescence spectrum of quantum dots exhibiting red shift to a longer wavelength dependent on the increase in temperature from 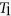 to 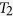. A ratio of fluorescence intensity of the lower area than the wavelength 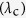 and the higher area of 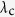 also elevates in accordance with temperature increment (from 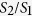 to 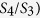. (**b**) Schematic diagram of the experimental set-up (see Methods “Ratiometry”). (**c**) Representative fluorescence spectrum of medium containing 50 nM quantum dots. Spectrum was separately acquired by PMT with 2 nm resolution (black dots). Experiments were performed at 27 °C. Gray curve indicates the fitted Gaussian function (mean: 651.0, sigma: 11.1). (**d**) Representative fluorescence images of quantum dots at 630–650 nm (upper) and 650–670 nm (lower) indicated by red lines in c. Scale bar represents 100 *μ*m. (**e**) Temperature dependency of fluorescence intensity ratio. Measurements were performed at 27 °C and 47 °C. Error bars represents s.e.m.

The maximum emission wavelength of quantum dots exhibits red shift dependent on the temperature^26^ with a sensitivity of 0.105 nm/°C^25^. But this sensitivity means that detecting 1 °C or less difference requires a resolution of about 0.1 nm. Moreover, the acquisition of the fluorescence spectrum takes too long time to measure the spatial or temporal temperature profiles. We focused on the red shift of quantum dots and examined whether or not a ratio of fluorescence intensity lower an wavelength 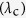 and that higher 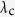, which can be resolved by an ordinary spectroscopic system, has dependency on temperature (Fig. 1a). The fluorescence intensity ratio was acquired by splitting the emission spectrum of a single quantum dot with a monochromator and by detecting them separately with a photomultiplier tube (PMT). We set up an experimental system for the ratiometry, using a confocal laser scanning microscope (FV1000, Olympus). Quantum dots were excited by 405 nm excitation laser. Ambient temperature was controlled and monitored with a stage-top incubator and a thermocouple (experimental set-up is shown in Fig. 1b).

Surface modified quantum dots, Qtracker Cell Labeling Kit (Q25021MP, Molecular Probes) are known to be incorporated into various cell lines thorough an endocytotic process^27^,^28^. A human derived neuronal cell line, SH-SY5Y, also endocytosed quantum dots after neuronal differentiation^29^. We prepared the 50 nM medium solution of this quantum dot crystals, and measured its fluorescence spectrum with 2 nm resolution of wavelength and 40× objective (Fig. 1c). Fitting the spectrum by Gaussian function, its mean value was calculated to 651.0 nm. When 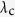 was set to 650 nm (near the mean value) and acquiring band widths of wavelength to 20 nm, the fluorescence intensity ratio (fluorescence intensity in 650–670 nm divided by that in 630–650 nm) exhibited significant difference between 27 °C and 47 °C (Fig. 1d,e). Thus we concluded the ratio 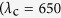 nm) as thermosensitive parameter.

### Fluorescence intensity ratio of single quantum dot as a thermosensitive parameter for time course analyses

**Figure 2:**
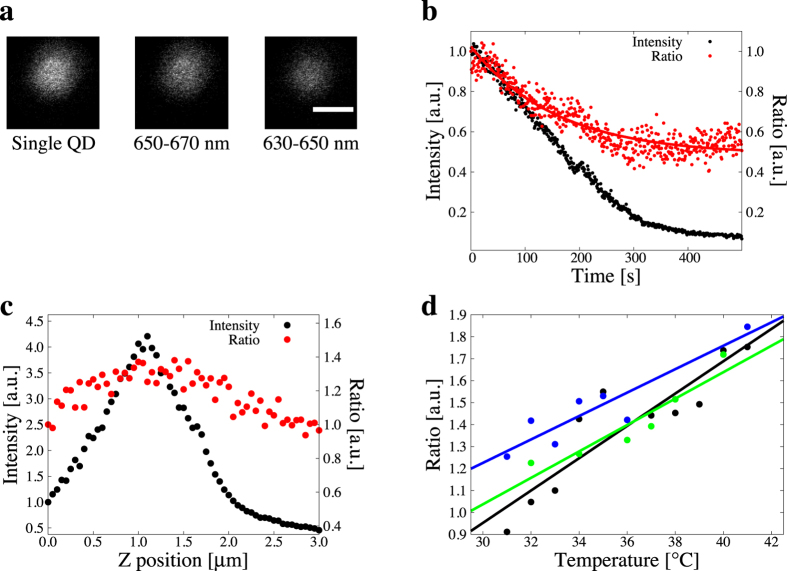
Temperature-dependent fluorescence intensity ratio of a single quantum dot. (**a**) Confocal fluorescence images of a single quantum dot. Left: an image of a quantum dot with the whole emission spectrum; middle: an image with the emission spectrum at 650–670 nm; right: an image with the emission spectrum at 630–650 nm. Scale bar represents 0.5 *μ*m. (**b**) Effect of photobleaching and heat storage on the fluorescence intensity ratio. Total fluorescence intensity and fluorescence intensity ratio were measured at every 1 s (normalized by their values at 0 s). The black and red dots indicate the fluorescence intensity and the ratio, respectively. Red line is the fitting curve of the ratio described in equation (2). (**c**) Relation between z-position and the fluorescence intensity ratio of single quantum dot. Focal plane was decremented by 0.05 *μ*m. Fluorescence intensity and fluorescence intensity ratio were normalized by their values at 0 *μ*m. (**d**) Correlation between ambient temperature and the fluorescence intensity ratio of single quantum dot. Distinct three single quantum dot were examined on the temperature sensitivity (shown in different color dots). Slope is 0.074/°C and 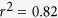 (black). Slope is 0.053/°C and 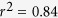 (blue). Slope is 0.060/°C and 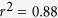 (green). Mean slope is 0.062/°C.

In our experiments the number of quantum dots incorporated into SH-SY5Y cells was kept low as they could be detected as independent particles. We performed the measurements of thermometry with each single quantum dot. In order to quantify the resolution and sensitivity of single quantum dots to temperature, we prepared a medium containing 1 nM quantum dots and performed the ratiometry. Both fluorescence intensity at 630–650 nm and 650–670 nm were captured with 100× objective (Fig. 2a). We first confirmed the effect of photobleaching on the fluorescence intensity ratio. In repeating the measurements, single quantum dot photobleached and the fluorescence intensity decreased as well as the fluorescence intensity ratio (Fig. 2b). The photobleaching curve of the fluorescence intensity ratio was fitted by an exponential function (eq. (2)). In following measurements the fluorescence intensity ratios were all calibrated by the function. The uncertainty of the fluorescence intensity ratio was also estimated from the time-lapse measurements (Fig. 2b) and it was 0.098 for 95% confidence interval. Thus we determined the temperature resolution of our methods as 0.098.

In our optical set-up (Fig. 1b), each quantum dot keeps moving around the focal plane by the thermal expansion of equipments and by Brownian motion of the particle, and it contributes to the focal position deviation and to the dispersion of fluorescence intensity ratio. We measured the fluorescence intensity ratio as changing the z position of microscope stage. Both the fluorescence intensity and the ratio were decremented along with the focal plane was close to the center of the particle (Fig. 2c). In the range of peak position ±0.25 *μ*m the fluorescence intensity ratio was not changed, so we determined to use the sample only in this range for following experiments.

We measured the fluorescence intensity ratio of a single quantum dot at 31–41°C which was controlled by a stage-top incubator. In all samples the fluorescence intensity ratio increased as the ambient temperature in the incubator was significantly elevated in this temperature range (Fig. 2d). The mean slope in this measurement was 0.062/°C and this is the temperature sensitivity of the fluorescence intensity ratio for our thermometry. Consequently, we confirmed the ratio as the relevant parameter to describe the correlation between temperature and the change of fluorescent peak drift. At the same time, photobleaching and the fluctuation of heating with the stage and chemical parameters^30^ do not correlate with the change of fluorescent peak drift. Reversibility of the fluorescence intenisity ratio was also examined as discussed in the past^31^,^32^,^33^. When cycling the measurements of *R* between two different temperatures, *R* did not changed (Supplementary Fig. S1). Thus the fluorescence intensity ratio was reversible.

Next we applied our thermometry to the living cell system. SH-SY5Y cells incorporated quantum dots and quantum dots localized in cytoplasm (Supplementary Fig. S2). We researched the temperature sensitivity of the fluorescence intensity ratio inside living cells. Single quantum dot in cytoplasm exhibited the positive correlation between the fluorescence intensity ratio and temperature (Supplementary Fig. S3). The slope was 0.067/°C and it can be compatible to the result outside cells (Fig. 2d), meaning that the temperature sensitivity of the fluorescence intensity ratio shows the robustness to the surroundings such as cytoplasma and culturing media.

### Measurement of thermogenesis in mitochondria

**Figure 3:**
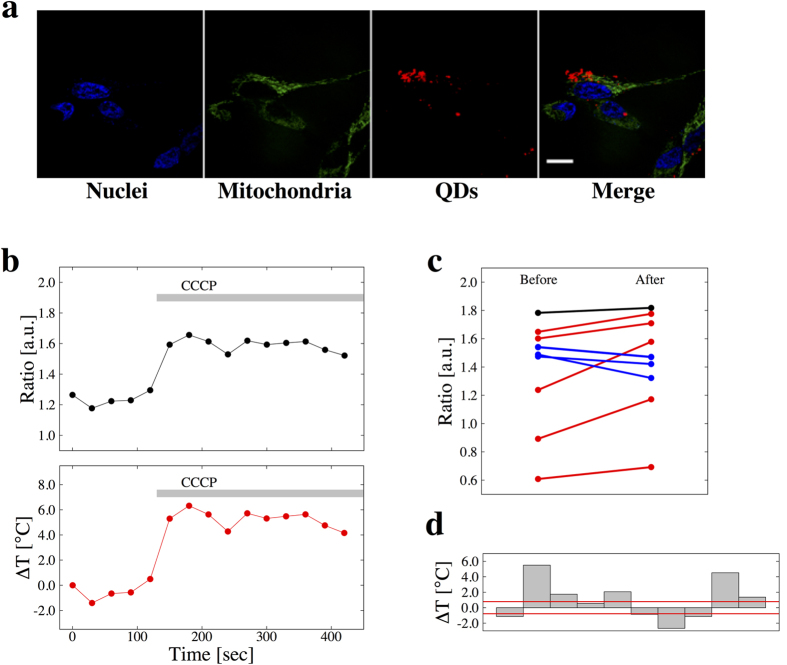
Quantification of heat production in mitochondria. (**a**) Confocal fluorescence images of living SH-SY5Y cells labeling nuclei by Hoechst 33342 (blue) and mitochondria by MitoTracker Green FM (green) and incorporating quantum dots (red). Scale bar represents 20 *μ*m. (**b**) Representative time-lapse of fluorescence ratio (upper) and the converted temperature change (lower) of a single quantum dot. Chemical stimulation by 10 *μ*M CCCP was applied at 130 sec. (**c**) Summary of fluorescence ratio response before (left) and after (right) CCCP stimulation. 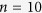. The number of samples of which the change in fluorescence intensity ratio before and after the stimulus was higher than the resolution and increase (red), in the range of the resolution (black), and higher than the resolution and decrease (blue) was 5, 1, and 4, respectively. Fluorescence intensity ratios were calibrated by estimated diameter. (**d**) Summary of temperature change for each measurement. Red lines present the resolution of temperature.

In order to confirm the validity of our temperature-sensing method in living cells, we measured the heat production in mitochondria. Carbonyl cyanide 3-chlorophenylhydrazone (CCCP), a protonophore and an uncoupler of oxidative phosphorylation, is known to accelerate the thermogenesis in mitochondria^34^. We applied 10 *μ*M CCCP to undifferentiated SH-SY5Y cells labelling mitochondria and incorporating quantum dots (Fig. 3a), and measured *R* of each quantum dots near mitochondria before and after the stimulation with CCCP.

Calibrated *R* values of single quantum dots near mitochondria were stable in large part during observation, while they tended to be high after the stimulation with CCCP (Fig. 3b). Although the *R* for each quantum dot has large variance, its behaviour on temperature change is comparable (Fig. 3b). Taking into account the resolution of *R* (Fig. 2d), *R* values were increased (out of the range in resolution) after the application of CCCP in large part (Fig. 3c,d). The mean temperature change was 0.94 °C and these observations were comparable to the previous work^15^. SH-SY5Y cells were also stimulated by DMSO. Then the temperature change with CCCP was larger than that with DMSO. Therefore, we concluded that the fluorescence intensity ratio has ability to detect the temperature change in living cells.

### Temperature difference in different compartments of neuronal cells

We investigated the existence of a spatial difference of temperature in a neuronal cell. For this purpose, we first evaluated the neuronal differentiation of SH-SY5Y cells in our experiment. SH-SY5Y cells were treated with 13-*cis* retinoic acid (RA) for 14 days to differentiate them^35^. To confirm the differentiation, neuron-specific class III *β*-tubulin (Tuj1)^36^ and intermediate filament-associated protein Nestin^37^ were labeled with antibodies conjugated with fluorophore. Tuj1 as the differentiated marker and Nestin as the undifferentiated marker were detected by a confocal microscope (Supplementary Fig. 4a). More quantitatively, both the RA treated cell population (RA_+_) and the non-treated cell population (RA_−_) were analyzed by flow cytometry. RA_−_ exhibited a higher fluorescence intensity of Nestin than RA_+_, and RA_−_ exhibited a lower fluorescence intensity of Tuj1 than RA_+_ (Supplementary Fig. 4b,c). We therefore concluded that RA_+_ cells were differentiated enough to have functions as neuronal cells.

**Figure 4:**
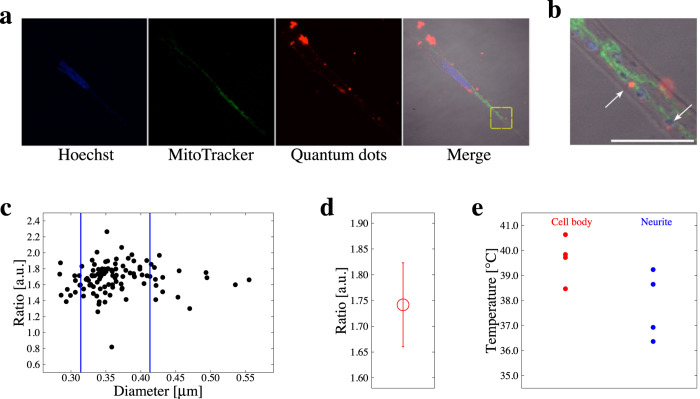
Temperature difference in neuronal cells. (**a**) Confocal fluorescence images of nuclei (blue), mitochondria (green), quantum dots (red), and the merge of fluorescence and DIC images. (**b**) A zoomed image around single quantum dot showed as yellow box in (**a**). White arrows point to the single quantum dot. Scale bar represents 10 *μ*m. (**c**) The relation between the estimated diameter of quantum dots and the fluorescence intensity ratio. The two blue vertical lines represent the thresholds of the estimated diameter at mean ± s.d. (0.314 and 0.413 *μ*m). The samples between the two thresholds were used for further analysis. (**d**) Variance in fluorescence intensity ratio of single quantum dot at certain temperature. (**e**) Statistically summarized results of thermometry. The mean temperature difference between the cell body and a neurite was 1.6 °C. *p-value* = 0.062.

Quantum dots were incorporated into RA_+_ cells and each *R* value of a quantum dot in the cells was measured by our ratiometry. In order to determine the position of quantum dots, nuclei and mitochondria were stained (Fig. 4a). In SH-SY5Y cells, each region of interest as a single quantum dot which had a Gaussian luminescence distribution and thus differed from aggregated particles was selected for measurements (Fig. 4b). Although quantum dots are known to exhibit a size-dependent spectral shift^38^, no significant size-dependency was shown in our experiments. However, some *R* values did not follow the temperature-dependency, and it was conspicuous in a sample with larger diameter which was estimated from the fluorescence image. Thus we set two diameter thresholds; the upper and lower thresholds were the mean ± s.d., and only the quantum dots which had their diameter between the thresholds were analyzed further (Fig. 4c). These samples had a variance in *R* at certain temperatures (Fig. 4d) and the 90% confidence interval was 0.16. This value was larger than the resolution of *R* and so 0.16 is the resolution of *R* for our spatial temperature measurement.

The quantum dots measured were separated into 2 groups in terms of the compartments they existed in, that is the cell body and the neurites. The mean *R* value of the sample from the cell body was 1.74 and that from neurites was 1.64 (Fig. 4e). The mean *R* difference was 0.10 (*p-value* = 0.062). This result means that the temperature in the cell body was 1.6 °C higher than that in neurites. Therefore, our thermometry detected the temperature difference in a neuronal cell.

## Discussion

In this study, we have constructed a method for measuring the intracellular temperature difference in a cell, which is based on commercially available quantum dot nanocrystals as the thermosensor and single particle spectroscopy as the principle of temperature detection. We developed two types of measuring strategies, one is for time course detection of temperature by using single particle, another is for detecting spatial distribution of intracellular temperature by taking the images of multiple quantum dot at the same timing. In both cases, we used the fluorescence intensity ratio.

In a previous work using quantum dots as a thermosensor^4^, temperature was calculated from the emission wavelength at the maximum intensity. Whether it is possible to obtain precise results by this method strongly depends on the resolution of the detector because of its principle. Compared to the above conventional method, the advantage of using the fluorescence intensity ratio, which is equal to detecting the total shift of the emission wavelength, is that this strategy is not susceptible to depend on the resolution of the detector but can contribute to the easier detection of temperature using a monochromator with a resolution of only 1 nm wavelength.

The relative temperature sensitivity is useful to evaluate the performance of various thermometry, each of which uses distinct thermometric parameters such as intensity, intensity ratio, and wavelength^39^. The relative temperature sensitivity (*S*) is defined as:


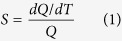


where *Q* corresponds to the variation of a thermometric parameter with temperature (*T*). In our method, thermometric parameters are the fluorescence intensity ratio and the maximum value of *S*(*S_m_*), which was 6.3%/K on average in Fig. 2d. In the conventional method using quantum dots^25^, *S_m_* is approximately 0.016%/K, so our method can improve the relative sensitivity. The limitation of the resolution of our temperature detection system is dependent on the optical resolution (approximately 200 nm), because the size of the quantum dot is smaller (10–20 nm) than the limitation of optical resolution. Temperature spatial resolution is provided in a previous work^15^ and the value of conventional infrared thermography is the order of 10 *μ*m^39^. Although in our method it is difficult to determine the spatial resolution in this definition because each quantum dot particle is not solute and is a smaller particle than the optical resolution, at the same time the frequency of quantum dot per volume was sparse for keeping the independency of each fluorescence, it can be comparable to our spatial resolution in terms of optical resolution (200 nm). Calorimetry is also one of the useful method to assess the temperature in living cells but it can only detect the heat production at single cell level^40^. By using a higher-resolution optical system such as a super-resolution microscope, the spatial resolution of our system will be improved.

We evaluated the temperature sensitivity of fluorescence intensity ratio of a single quantum dot both in media and a cellular condition. This is common research because the temperature sensitivity of thermometer can be altered by the environmental condition such as protein concentration, pH, or ionic strength^16^,^19^. Our evaluation indicated that the fluorescence intensity ratio of single quantum dot exhibited the similar sensitivity, so our methods is applicable to the temperature detection regardless of the cellular condition or not.

Our experimental results demonstrate a large variance of *R* both outside (Fig. 2) and inside (Figs 3 and 4) the cells. The cause of this variance is thought to be the combined factors of followings: 1) an uncertainty of the fluorescence intensity ratio of single quantum dot (Fig. 2b), 2) a variance of the fluorescence intensity ratio in distinct quantum dots (Fig. 4d), 3) the distance between quantum dots where thermometry was executed and certain organelles as a heat source^4^, and 4) a heterogeneity in temperature in cytoplasm^5^,^21^. We eliminated the factor (2) by excluding some erratic samples which had a larger or smaller estimated diameter than major samples of diameter of quantum dot (Fig. 4c). The factor (1) was possibly caused by the resolution of the monochromator or the precision of the temperature controlling devices. In our method, the factor (1) will diminish by using higher-performance devices and the accuracy was to some extent assured by the number of trials in order to detect the temperature difference.

We performed time course measurements with single quantum dot, and analysed spatial distribution with multiple quantum dots, separately. By checking the precision of our method based on the single quantum dot measurement, we found that the divergence of temperature-ratio correlation lines was mainly proceeded from the difference of individual character of each quantum dot, which appeared as their intercepts of correlation lines. It was clear that the correlation between temperature and ratio was strong (correlation coefficients *R* are 0.91 ~ 0.94) when we calculate the correlation line for each quantum dot despite if we merge all the data produced with different quantum dots, the correlation of temperature and ratio was moderate (*R* = 0.61). In order to avoid the effect of the difference among each quantum dot, instead of merging the data points of different quantum dots, we calculated correlation lines for each quantum dot and took their mean of the slope values.

We evaluated our originally established method for thermometry by using well-analysed cell biological heating phenomena (Fig. 3). CCCP treatment is expected to an elicit increase in intracellular temperature^15^. Our results exhibit a large variance in *R* as discussed above. This happens mainly because the ratio of quantum dots has variance which is originated in the variance of each quantum dot (Fig. 2d). The difference in *R* still existing of before and after the application of CCCP indicates the acceleration of thermogenesis in mitochondria. The difference in temperature before and after the stimulation (Fig. 3c) was not significant. This is because there exists a temperature distribution in mitochondria^15^ and the distance between quantum dots and mitochondria cannot be completely controlled. We thus concluded that our originally established thermometry method is sufficient to analyze the intracellular upregulation of temperature at the physiological environment.

For the purpose of researching the temperature difference in neuronal cells, we conducted our established thermometry with SH-SY5Y cells. The *R* difference between a cell body and neurites showed 0.10 corresponding to a few degree based on the calibration line (Fig. 2d). This result is reasonable as the distribution of an intracellular temperature distribution by comparing its range with the previous works about the temperature detection of different organelle. We confirmed this by producing a range of confidence intervals for the slope. By applying the steep line in the range of confidence intervals to calculate the temperature, it is possible for the difference in absolute temperature is possible to be 1 °C only. In order to guarantee the quantitative significance of the results, we will need to eliminate the variance of *R*, as stated above.

Our thermometry reveals for the first time the heterogeneity of temperature in the cytoplasm of neuronal cells. However, the existence of an intracellular temperature distribution is controversial^41^. In order to explain the temperature distribution in such a small region as the cell body and neurites, we considered two hypotheses. One is that their characteristic shape can contribute to the difference in ability to store heat. To quantify their ability to store heat, we calculated the specific surface area of the cell body and neurite from z-stack fluorescence images. The results of the experiments showed that the surface area of a neurite is approximately 1.3 times larger than that of cell body. This result implies that a neurite dissipates heat more easily and corresponds more closely to ambient temperature than the cell body. At the same time, cell body includes a particular heat source, nucleus, which is not involved in neurites. It was observed that the temperature of a nucleus is about a degree higher than that of the cytoplasm^5^. This fact can be the cause to maintain the temperature of cell body high by retaining the produced heat. To build a clearer hypothesis, it will be efficient to perform a simulation of heat conduction by using the actual z-stack images of cells.

Surface-volume ratio is an index which can be calculated the quantitative values of area and volume of the objects, which are the direct quantities to estimate further cause of thermal storage or dissipation, and seems it has a measurable significance. Now we got the other question that if the shape of the object itself is also significant or not. In this point of view, surface-volume ratio is not always appropriate to appreciate it. We analysed another index to figure out the difference between the shape of a cell body and a neurite. Sphericity is an index to compare the roundness of a 3-dimensional (3D) object. If the value equals 1.0, that means the 3D object is a perfect sphere, if the shape of 3D object is discrete from a sphere, the value is close to 0. We calculated this value of the cell body region and the neurite region as the first step to compare the characteristics of their shape. The sphericity of the cell body was 0.34, the value of the neurite was 0.28. The ratio of sphericity between the value of the cell body 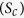 and the neurite 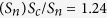. There exists positive correlation between the temperature and the sphericity within the limits of our analysis until now. We need further investigation to get conclusion with more detailed analyses of the shape of each target compartment.

Another hypothesis is that the types of reactions occurring in the cell body and in the neurites are different in order to generate different functions, and this causes the difference in the amount of heat production. Several previous works have shown a temperature increase inside a cell following the influx of Ca^2+^ ion^4^,^11^,^13^,^18^. The localized influx of Ca^2+^, for example in synapses, can cause the temperature difference inside a neuron. This localization of reactions dependent on their types can be seen in previously established long-term memory models. Our previous work showed that reaction-limited reactions mainly exist in the cell body and diffusion-limited reactions are localized in neurites^42^. However, the reaction-limited system is in principle more difficult to be affected by temperature; this means that a higher temperature in the cell body is probably little affected for generating functions, and vice versa.

This paper shows that temperature difference does exist in micrometer scale domains in a cell. Although mapping temperature spatiotemporally will continue to be challenging future work, the methodology is in the process of being sophisticated. Recently a technique of heating cell locally by using IR laser has also been developed^43^,^44^,^45^. Furthermore, a theoretical approach to understanding the cell dynamics such as microtubule shrinkage has been reported^46^. These techniques are all useful to study not only unknown mechanisms related to proteins working as a heat source but also various behaviors related to heat production such as energy metabolism, apoptosis or neoplastic transformation.

## Methods

### Quantum dot samples

Quantum dots Qtracker 655 Cell Labeling Kit (Q25021MP, Invitrogen) was used for all following measurements. Qtracker nanocrystals and Qtracker carrier were mixed and incubated for 5 min at room temperature to prepare the 1 *μ*M solution. The solution was diluted with 37 °C cell culture medium for the concentration of 10 nM or 50 nM and vortexed for 30 seconds.

For the measurements of single quantum dot outside cells, 10 nM solution was further diluted to 1 nM.

### Ratiometry

For ratiometry, a confocal laser scanning microscope and its software (FV1000, Olympus) and 40× (numerical apeture (N.A.) 1.3) or 100× (N.A. 1.4) objective were used. Ambient temperature near the region of interest was monitored with a thermocouple (5SC-TT-K-36-36, Omega Engineering) and Arduino (ARDUINO-A000057, Switch Science) and controlled with a stage-top incubator (INUG2-ONICS, Tokai Hit). Diluted quantum dot solution was mounted on the 35 mm glass base dish and placed in the stage-top incubator set on the microscope stage. Quantum dots were excited by 405 nm laser, then its emission spectrum was dispersed in the range of 630–650 nm and 650–670 nm by a monochromator. The two emission spectra were collected by PMT and imaged with software. Total fluorescence intensity in the region of interest for 650–670 nm was divided by that for 630–650 nm and the value (fluorescence intensity ratio) was defined as the thermometric parameter.

For the acquisition of fluorescence spectrum of 50 nM quantum dots solution, 405 nm laser was applied to the sample. The emission in the range of 600–700 nm was split every 2 nm and detected by PMT. Acquired spectrum was fitted by Gaussian function and the mean value was estimated.

### Evaluation of fluorescence intensity ratio of single quantum dot

100× oil objective (N.A. 1.4) was used. To evaluate the photobleaching effect, fluorescence intensity ratio of single quantum dot was repeatedly acquired every 1 sec, 500 times. Photobleaching curve was fitted by the function:


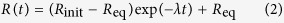


where 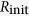 and 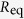 are the initial value and the equilibrium value of the fluorescence intensity raito, respectively, and *λ* is the decay constant. As a result the decay constant was determined as 0.00635 s^−1^. In order to get the z profile of fluorescence intensity ratio, z-Stack image acquisition for single quantum dot was performed with FV1000. Focal plane was decremented by 0.05 *μ*m. Acquired fluorescence intensities and fluorescence intensity ratios were calibrated by the photobleaching curve (eq. (2)) shown in Fig. 2b. To evaluate the temperature sensitivity of single quantum dot outside the cells, the fluorescence intensity ratio was measured at each temperature, which was changed by 1.0 °C in a range of 30–42 °C. The reversibility of the fluorescence intensity ratio to the temperature was examined by the repetitive aquisition at 40 °C and 30 °C controlled by a stage-top incubator.

### Cell culture and differentiation

We obtained a SH-SY5Y cell line from the European Collection of Cell Cultures (ECACC) Cell Bank; the cell line Cat. No. is 94030304. Cell culture reagents for SH-SY5Y cells were obtained from Wako Pure Chemical Industries Ltd (Osaka, Japan).

The cell line was cultured routinely in Dulbecco’s Modified Eagle MediumNutrient Mixture F-12 (DMEMF-12) supplemented with 15% fetal bovine serum (FBS) in 5% CO_2_ incubator at 37 °C. In all experiments with a microscope, cells were subcultured in a 35 mm glass base dish (IWAKI) coated with 150 *μ*g/mL collagen (Cellmatrix Type IV, Nitta Gelatin). For neuronal differentiation, 13-cis retinoic acid (R3255, Sigma) was added to the culture medium at 10 *μ*M every 2 days for 14 days.

### Temperature measurements in living cells

The culture medium in the 35 mm glass base dish with living SH-SY5Y cells was replaced with 10 nM quantum dot solution and incubated for 60 min in a 5% CO_2_ at 37 °C. Then the cells were washed with culture medium and used for measurements. In the measurements, temperature were kept 37 °C by a stage-top incubator. All the ratiometry in living cells were performed with 100× (N.A. 1.4) objective. To evaluate the temperature sensitivity of single quantum dot inside the cells, cells were labelled with Hoechst 33342 (PA-3014, Lonza). The fluorescence intensity ratio was measured at each temperature, which was changed by 1.0 °C in a range of 30–40 °C. To measure the thermogenesis in mitochondria, cells were labelled with MitoTracker Green FM (M7514, Molecular Probes) and Hoechst 33342. 10 *μ*M CCCP (C2759, Sigma) as a chemical stimulus was applied to the cells. The fluorescence intensity ratio of single quantum dot near mitochondria was acquired every 30 seconds before and after the stimulation.

For the acquisition of fluorescence images of SH-SY5Y cells, confocal laser scanning microscope (FV1000, Olympus) and 40× (N.A. 1.3) or 100× (N.A. 1.4) objective were used. DNA labelled with Hoechst 33342, mitochondria labelled with MitoTracker Green FM and quantum dots were excited by a 405 nm laser or a 488 nm laser, and the images were captured through dichroic mirrors, emission filters and PMTs.

### Immunostaining

SH-SY5Y cells were fixed with 4% formaldehyde (064–00406, Wako) for 15 min at room temperature, and then washed and incubated in PBS containing 0.1% Triton X-100 (A16046, Alfa Aesar) for 10 min at 4 °C for permeabilization. The cells were incubated in PBS containing 1% BSA (017–22231, Wako) for 30 min at 4 °C for blocking. Primary antibodies and secondary antibodies were incorporated and then the cells were incubated overnight at 4 °C and for 60 min at room temperature. After the antibodies had been removed, the nuclei were stained with 0.1 *μ*g/mL Hoechst 33342 (PA-3014, Lonza) for 5 min at room temperature.

The following combination of primary and secondary antibodies diluted by 1% BSA in PBS was used: a rabbit monoclonal anti-Nestin (ab105389, 1:200, Abcam) and a donkey anti-rabbit-IgG(H+L) conjugated with Alexa Fluor 594 (a21207, 1:1000, Invitrogen), and a mouse monoclonal anti-Tuj1 (mms-435p-100, 1:1000, BioLegend) and a donkey anti-mouse-IgG(H+L) conjugated with Alexa Fluor 488 (a21202, 1:1000, Invitrogen).

405, 488 and 559 nm continuous wave lasers were used to excite the fluorescent dye through a confocal microscope (FV1000, Olympus). An objective (40×, N.A. 1.3) was used to acquire the spectra. Each emission of fluorescent dye was collected through dichroic mirrors and an emission filter, and imaged by a PMT.

### Flow cytometry

To validate the neuronal differentiation of SH-SY5Y cells, retinoic acid-treated cells were analyzed by flow cytometry. SH-SY5Y cells were fixed with 4% formaldehyde for 15 min at 4 °C, and then washed and permeabilized with 0.1% Triton X-100 for 15 min at 4 °C. The cells were incubated with primary antibodies for 30 min at room temperature, and then incubated with the corresponding secondary antibodies for 30 min at room temperature.

The combination of primary and secondary antibodies diluted by 3% BSA in PBS was the same as that used in the immunostaining. The dilution ratio was as follows: anti-Nestin (1:50), anti-rabbit-IgG (1:500), anti-Tuj1 (1:100), anti-mouse-IgG (1:500).

The cells were analyzed with a flow cytometer (Epics XL, Beckman Coulter). Fluorescent dyes were excited with an Ar Laser beam and their emission spectra were collected through a 525 band path filter or 620 band path filter into each PMT. All the experimental results were analyzed with a flow cytometry data analysis tool (FlowJo, http://www.flowjo.com).

### 3D image analysis

Surface area, volume and sphericity of cell compartment were calculated using ImageJ 3D Shape plugin.

## Additional Information

**How to cite this article**: Tanimoto, R. *et al.* Detection of Temperature Difference in Neuronal Cells. *Sci. Rep.* **6**, 22071; doi: 10.1038/srep22071 (2016).

## Supplementary Material

Supplementary Figures
