# Supplementary material for: Detection of Temperature Difference in Neuronal Cells
Source: Sci Rep. 2016 Mar 1;6:22071. doi: 10.1038/srep22071 (PMC4772094; doi:10.1038/srep22071)
Supplement: Supplementary Figures [file srep22071-s1.pdf]

Supplemental Data of

Detection of Temperature Difference in Neuronal Cells

Ryuichi Tanimoto[1], Takumi Hiraiwa[1], Yuichiro Nakai[1], Yutaka Shindo[1], Kotaro Oka[1], Noriko Hiroi[1,*], and Akira Funahashi[1]

[1]Keio University, Department of Biosciences and Informatics, 3-14-1, Hiyoshi, Kohoku-Ward, Yokohama, 223-8522, Japan
[*] hiroi@bio.keio.ac.jp

contents:

Cover page

page 1

Figure S1

Figure S2

page 2

Figure S3

page 3

Figure S4

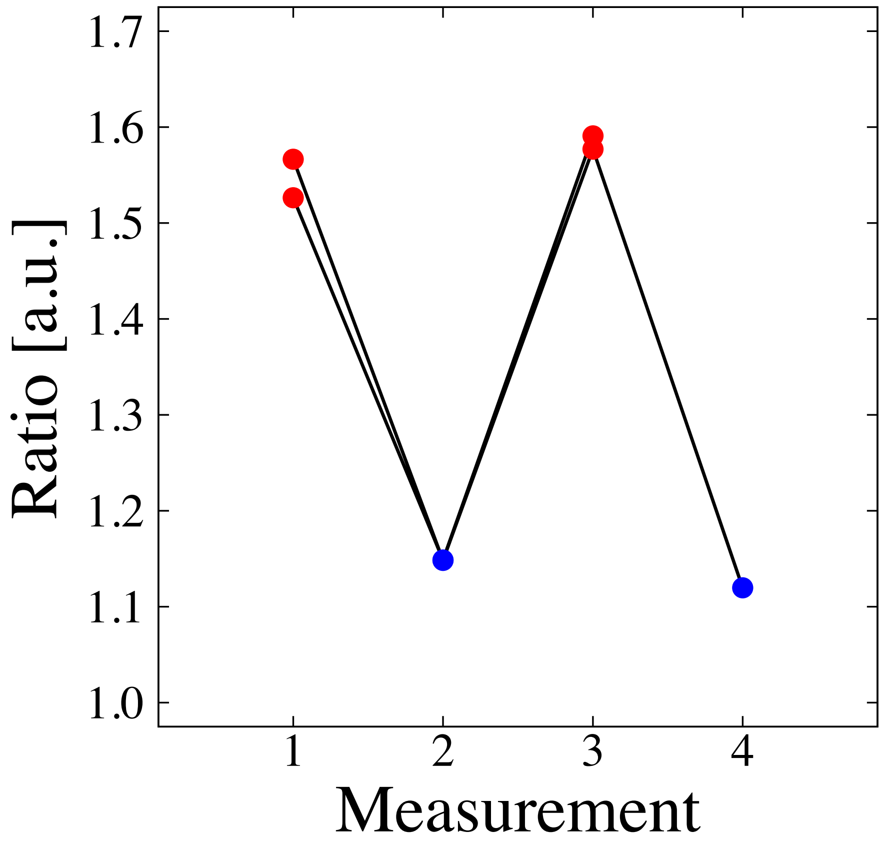

Figure S1. Representative reversibility of the fluorescence intensity ratio of quantum dots to temperature. Red and blue dots indicate the mean value of fluorescence intensity ratio at 40°C and 30°C. All plots were calibrated by the photobleaching curve.

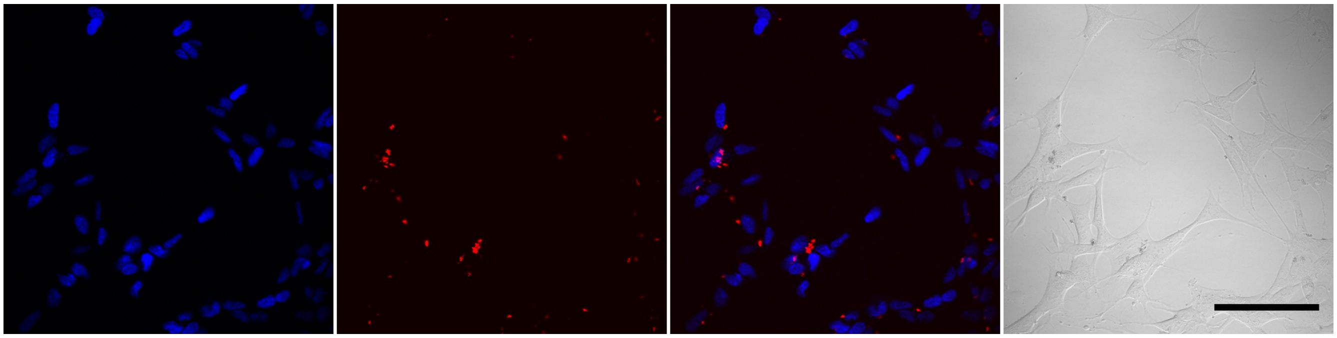

Figure S2. Fluorescence images of SH-SY5Y cells incorporating quantum dots. Blue: DNA. Red: Quantum dots. Most right is the phase contrast image. Scale bar represents 100 μm.

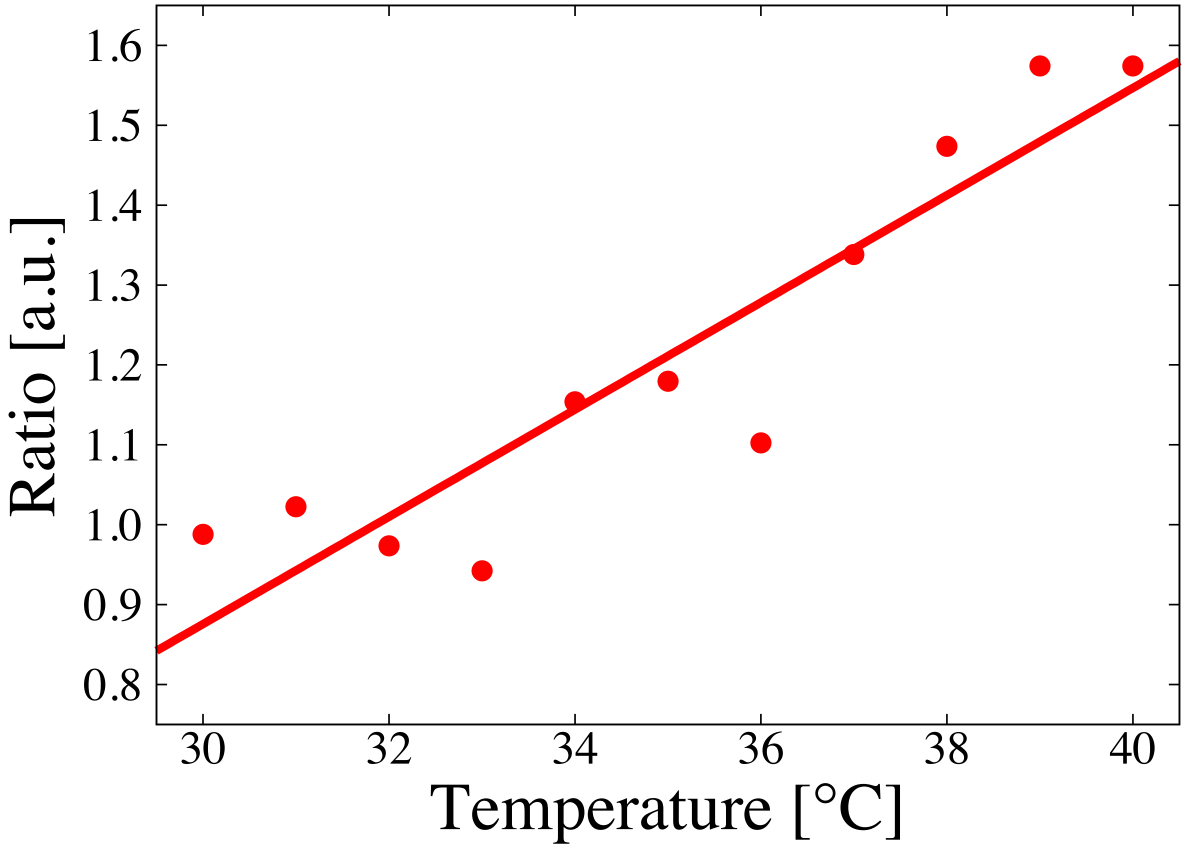

Figure S3. A correlation between the fluorescence intensity ratio of single quantum dot inside SH-SY5Y cell and ambient temperature. Slope is 0.067/°C and $r^2 = 0.86$.

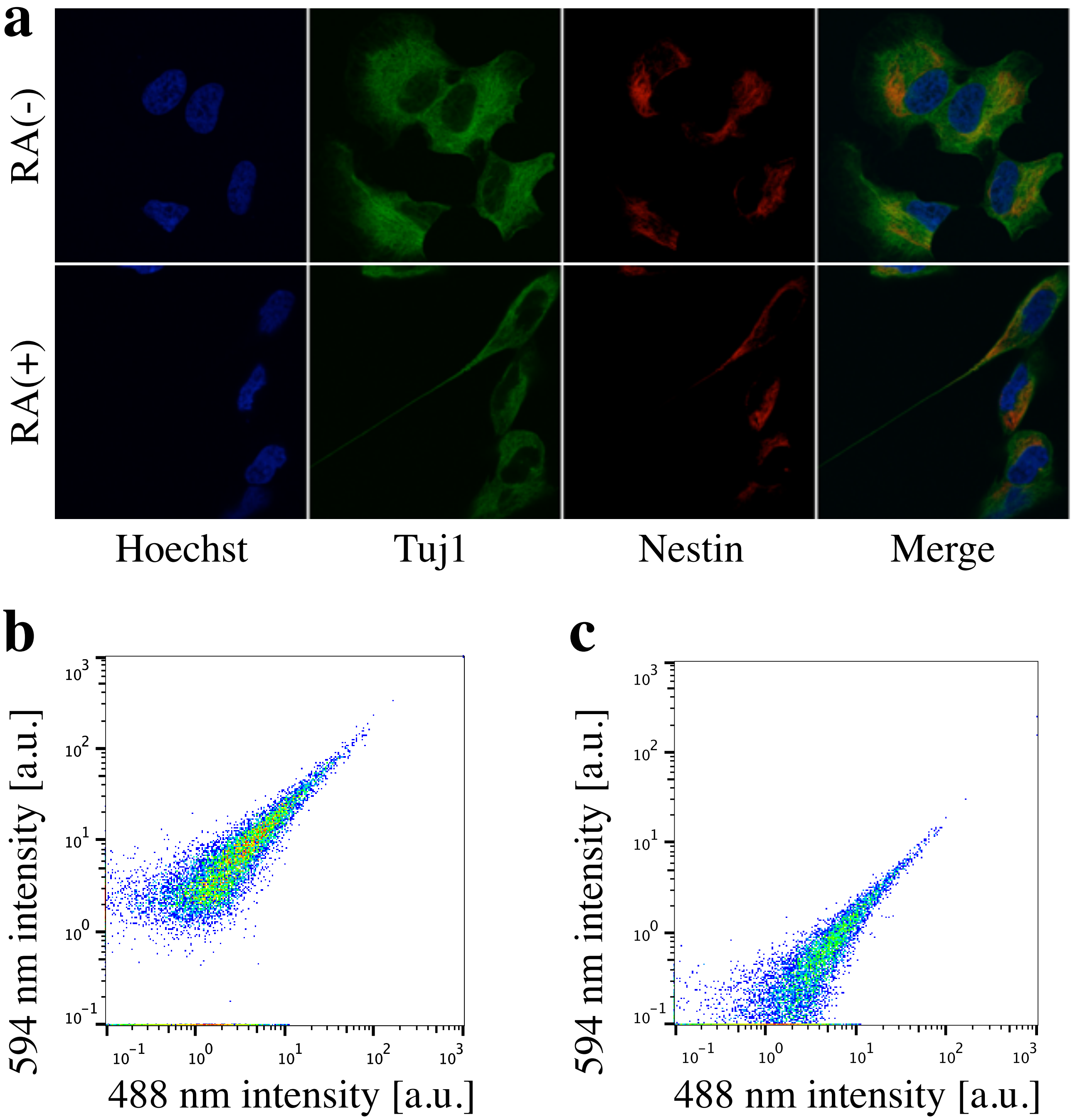

Figure S4. Neuronal differentiation of SH-SY5Y cells. (a) Confocal fluorescence images of SH-SY5Y cells before and after the treatment with 13-*cis* retinoic acid. Fluorescence of DNA (blue), Tuj1 (green) and Nestin (red) are shown. Scale bar represents 20 μm. (b, c) The expression profile of RA_{-} (b) and RA_{+} (c) analyzed by flow cytometry. The horizontal axis indicates the expression level of Tuj1, and the vertical axis Nestin.
